# Role of chromosomal instability and clonal heterogeneity in the therapy response of breast cancer cell lines

**DOI:** 10.20892/j.issn.2095-3941.2020.0028

**Published:** 2020-12-15

**Authors:** Natalia Vargas-Rondón, Erika Pérez-Mora, Victoria E. Villegas, Milena Rondón-Lagos

**Affiliations:** 1 School of Biological Sciences, Universidad Pedagógica y Tecnológica de Colombia, Tunja 150003, Colombia; 2 Biology Program, Faculty of Natural Sciences, Universidad del Rosario, Bogotá 111221, Colombia

**Keywords:** Breast cancer, chromosomal instability, therapy resistance, FISH, clonal heterogeneity

## Abstract

**Objective:**

Chromosomal instability (CIN) is a hallmark of cancer characterized by cell-to-cell variability in the number or structure of chromosomes, frequently observed in cancer cell populations and is associated with poor prognosis, metastasis, and therapeutic resistance. Breast cancer (BC) is characterized by unstable karyotypes and recent reports have indicated that CIN may influence the response of BC to chemotherapy regimens. However, paradoxical associations between extreme CIN and improved outcome have been observed.

**Methods:**

This study aimed to 1) evaluate CIN levels and clonal heterogeneity (CH) in MCF7, ZR-751, MDA-MB468, BT474, and KPL4 BC cells treated with low doses of tamoxifen (TAM), docetaxel (DOC), doxorubicin (DOX), Herceptin (HT), and combined treatments (TAM/DOC, TAM/DOX, TAM/HT, HT/DOC, and HT/DOX) by using fluorescence *in situ* hybridization (FISH), and 2) examine the association with response to treatments by comparing FISH results with cell proliferation.

**Results:**

Intermediate CIN was linked to drug sensitivity according to three characteristics: estrogen receptor α (ERα) and HER2 status, pre-existing CIN level in cancer cells, and the CIN induced by the treatments. ERα+/HER2**−** cells with intermediate CIN were sensitive to treatment with taxanes (DOC) and anthracyclines (DOX), while ERα**−**/HER2**−**, ERα+/HER2+, and ERα-/HER2+ cells with intermediate CIN were resistant to these treatments.

**Conclusions:**

A greater understanding of CIN and CH in BC could assist in the optimization of existing therapeutic regimens and/or in supporting new strategies to improve cancer outcomes.

## Introduction

The therapeutic decision regarding estrogen receptor-­positive (ERα+) and ERα-negative (ERα−) breast cancer (BC) patients is mainly based on the evaluation of clinicopathological characteristics. In particular, nodal status, tumor size, and lymph node status play a major role in the selection of therapeutic strategies including hormonal therapy and/or chemotherapy with different agents. However, although the evaluation of these parameters has allowed the survival of a large number of patients, some of them relapse and eventually develop resistance to treatment over time. Therefore, identifying reliable prognostic and predictive markers is a priority in BC research. A promising therapeutic target is chromosome instability (CIN), a common feature in solid tumors^1^. BC is characterized by unstable karyotypes and recent reports have indicated that CIN may influence response to distinct chemotherapy regimens in HER2-positive (HER2+) tumors^1–3^.

CIN is characterized by the gain or loss of partial chromosomes or whole chromosomes (aneuploidy)^3^. Aneuploidy refers to the state of abnormal chromosome number, which can be either stable or unstable. Unstable aneuploidy leads to karyotypic heterogeneity between tumor cells^4^, which in turn can lead to the simultaneous growth of diverse tumor sub­populations [clonal heterogeneity (CH)], resulting in genomic inter- and intra-tumor heterogeneity^3,5^. In addition, both CIN and CH have been associated with cancer progression, increased invasiveness, and response to ­therapy^6–8^. Karyotypes of breast tumors are characterized by a high grade of complexity with multiple chromosomes showing both numerical and structural changes. Alterations of chromosome arms 1q, 3p and +7, +8, +20 have been frequently observed in BC, together with cytogenetic signs of DNA amplification, such as homogeneously staining regions that are preferentially associated with 8p and ­17q^9–11^. Moreover, loss of heterozygosity (LOH) and comparative genomic hybridization (CGH) studies showed deletion and amplification of large genomic segments. Common regions of LOH in BC are located on several chromosomes, including 1p, 1q, 3p, 8p, 11q, 13q, 16q, 17p and 17q, while hot spots for gains are routinely observed at 1q, 8q, 11q, 17q and 20q^12^. Further, cytogenetic and molecular observations show that breast tumors are characterized by ­multiclonality, suggesting the existence of a high degree of intratumoral heterogeneity, mostly sustained by CIN. CIN and CH lead to gene regulatory interactions and varying protein concentrations, both of which could impact cell responses to drug treatments^13^. Some reports suggest that chromosomal alterations in individual cancer cells induce variable drug sensitivity and thus can lead to the survival of a fraction of tumor cells^14^. For instance, it has been reported that while HER2+ tumors with low CIN (relative karyotype stability) are associated with taxane sensitivity, tumors with high CIN (different patterns of karyotype complexity) are associated with therapy sensitivity based on anthracycline and platinum^15,16^. However, paradoxical relationships between extreme CIN and improved outcome have been observed in patients with various types of cancers, including BC^17,18^. In fact, high CIN has been correlated with improved long-term survival in ERα− BC patients, but poorer outcome in ERα+ BC patients^19^. These observations show the complexity associated with selecting appropriate therapies targeting CIN in different types of cancers and suggest that a threshold of CIN may exist that, when exceeded, could induce either cell cytotoxicity or cell survival. However, these CIN thresholds are theoretical and could be specific for each type of cancer.

Taking into account the high frequency of patients who do not respond to therapy or who develop resistance over time, determining and standardizing new prognostic and predictive markers is critical in BC research. Better understanding of the associations between the levels of CIN and CH with the response to therapy may help determine whether there is a critical CIN threshold that distinguishes tumor lethality from viability. These findings could allow us to predict the benefit of chemotherapy, hormone therapy and combined therapies in patients with BC, as well as to develop new strategies to improve the prognosis of cancer.

The aim of this study was to determine the CIN level and CH by using fluorescence *in situ* hybridization (FISH), in five human BC cell lines with differential expression of ERα and HER2 and to examine the association with the response to individual treatments, tamoxifen (TAM), docetaxel (DOC), doxorubicin (DOX), and Herceptin (HT), and combined treatments, TAM+DOC, TAM+DOX, TAM+HT, HT+DOC, and HT+DOX.

## Materials and methods

### Cell lines

The human BC cell lines MCF7 and ZR75-1 (ERα+/­progesterone receptor (PR)+/HER2−), MDA-MB468 (ERα−/PR−/HER2−), BT474 (ERα+/PR+/HER2+), and KPL4 (ERα−/PR−/HER2+) were obtained from the American Type Culture Collection (ATCC). Cell lines were expanded and stocked at −80 °C and cells obtained from these stocks were thawed and used for the experiments. To confirm the authentication of the cell lines, short tandem repeat profiles were performed at the end of experiments. All experiments were carried out in each cell line at passages (P) below 19. MCF7 (P8), ZR75-1 (P13), MDA-MB468 (P11), and KPL4 (P18) were cultured in Roswell Park Memorial Institute (RPMI)-1640 medium (Sigma, St. Louis, MO, USA), whereas BT474 (P17) was cultured in Dulbecco’s Modified Eagle’s Medium (DMEM) (Sigma). All culture media were supplemented with Antibiotic–Antimycotic Solution (100 ×) (Sigma), 10% fetal bovine serum (FBS) (Sigma) and L-glutamine (2 mM) (Invitrogen GmbH). Cells were cultured in 75 cm^2^ (10 mL) flasks at 37 °C and 5% CO_2_. The absence of contamination with mycoplasma was confirmed by polymerase chain reaction (PCR) assay.

### Treatments

BC cell lines were treated with TAM (T5648; Sigma), DOC ­(sc-201436; Santa Cruz Biotechnology, Dallas, USA), DOX (sc-200923; Santa Cruz Biotechnology), HT (L01 XC03; Roche, Basel, Switzerland) and combined treatments (TAM/DOC, TAM/DOX, TAM/HT, HT/DOC, and HT/DOX).

TAM, DOC, DOX, and HT were dissolved in absolute ethanol and diluted in media at 1 μM, 10 nM, 0.5 μM and 50 μg/mL, respectively, and then added to the culture medium for 24 h, 48 h, and 96 h. These concentrations have been demonstrated to be the highest and the best doses at which an effect (changes on the cytoskeleton architecture and cell death) in BC cells *in vitro* was observed^20–23^. Each drug and/or combination was added to the cell lines according to expression of ERα and HER2. Specifically, cell lines positive and negative for ERα were treated with hormonal therapy (TAM) and combination of TAM with chemotherapy (DOC and DOX), while HER2+ cell lines were treated with HT and combination of HT with chemotherapy (DOC and DOX).

Untreated cells were used as controls. Control cells were used with the same volume of culture medium and incubated together with experimental groups (drug treatment groups). The treatment strategy is indicated in the **Supplementary Table S1**.

### Proliferation assay

Cells were seeded at a density of 2.5–5 × 10^3^ cells per 100 μL of phenol red-free medium in a 96 multi-well plate. After 24 h, cells were treated with TAM, DOC, DOX, HT, and combined treatments (TAM/DOC, TAM/DOX, TAM/HT, HT/DOC, and HT/DOX) for 24 h, 48 h, and 96 h. At the end of each treatment, cell proliferation was assessed using the cell proliferation enzyme-linked immunosorbent (ELISA) kit, BrdU (Roche Diagnostics Deutschland GmbH, Mannheim, Germany). Measurement of absorbance was performed using a Tecan Infinite M200 reader (Tecan Trading AG, Männedorf, Switzerland) against a background control as blank. Each treatment was performed in 24 replicates. Data are expressed as means ± standard deviation (SD).

### Metaphase and nuclei spreads

To determine the induction of CIN and CH, we performed molecular cytogenetic analysis (FISH) on both control and treated BC cell lines. Metaphases were obtained using standardized harvesting protocols. Briefly, Colcemid Solution (0.03 μg/mL) (Sigma) was added to cultures 2.5 h before cell harvesting; cells were then treated with hypotonic solution, fixed 3 times with Carnoy’s fixative (3:1 methanol to acetic acid), and spread on glass. Metaphase and nuclei spreads were subsequently hybridized with centromere probes using FISH.

### FISH and CIN evaluation

CIN was evaluated on the metaphase and nuclei spreads obtained previously by FISH using six centromeric probes (CEP) for chromosomes 2, 3, 8, 11, 15, and 17 (all from Cytocell, Cambridge, UK) and standard procedures. Three-color FISH was performed on nuclei/metaphase spreads for chromosomes 2, 8, and 11 and chromosomes 3, 15, and 17 using centromeric probes labeled with different spectrum colors: spectrum orange for CEP2 and CEP3; spectrum aqua for CEP8 and CEP17; and spectrum green for CEP11 and CEP15. Chromosomes 2 and 15 were selected as these chromosomes presented infrequent copy number alterations in a series of breast tumors analyzed by microarray-based comparative genomic hybridization (aCGH) analysis^19^. Chromosomes 3, 8, 11, and 17 were selected as these chromosomes are ­frequently altered in BC^12^. Some studies have reported an association between alterations in chromosome 17 with sensitivity to anthracycline treatment^24^. Centromeric FISH is the most-commonly employed method to assess numerical CIN in tumoral cells^4,25–27^. The major benefits of this approach are that it allows hundreds of cells to be evaluated at a time, it allows accurate measurement of cell-to-cell heterogeneity within the tumor as well as allowing classifying and differentiating aneuploid tumors: those with high CH (unstable aneuploidy) and those with low CH (stable aneuploidy)^28–30^. In addition, some reports showed that the use of probes for just two chromosomes was sufficient to identify diploid from aneuploid tumors^31,32^. However, one advantage of using more than two probes is that clonal populations can be identified with greater certainty^33^.

Ten randomly selected areas of each BC cell line were acquired using an Olympus microscope with the cytogenetic software Cytovision System 7.4 (Leica Biosystems Richmond, Inc.). CIN was assessed in a minimum of 100 intact and non-overlapping nuclei and some metaphases for each chromosome. The CIN rate for each cell line was defined first by calculating the percentage of nuclei with a CEP signal number different to the modal number (the most common chromosome number in a tumor cell population) for each individual chromosome and then calculating the mean CIN percentage of all chromosomes analyzed^24,34^. According to the level of CIN, we classified the cell lines as having low CIN (CIN 0%–30%), intermediate CIN (CIN 31%–70%), or high CIN (CIN >70%).

Considering that the CIN may similarly classify stable aneuploid tumor cells with relatively few clones making up a large proportion of the tumor (e.g., 80% nuclei with two centromeres and 20% nuclei with three centromeres) together with unstable aneuploid tumor cells with high CH (e.g., 70% of nuclei with two centromeres, 20% with three, 5% with four, and 5% with five centromeric signals), we calculated the Shannon Diversity Index (SDI) to assess more directly the CH within each cell line before and after treatments. SDI integrates both the number and abundance of cell clones within each cell line according to published methods^19,35^.

### Statistical analysis

The CIN levels observed after treatments were determined in comparison with the control. Student’s *t*-test was performed to compare cell proliferation of treated cell lines with untreated cell lines. The CH within each cell line was determined by calculating the SDI, which integrates both the number and abundance of cell clones within each cell line according to published methods^19,35^. The SDI (H) was estimated for chromosomes 2, 3, 8, 11, 15, and 17 using the following formula:

$$H=-\sum_{i}p_{i}\ln(p_{i})$$

in which *p_i_* is the frequency of centromere signal, *i*^19,35^. The normal value of this *H* index is between 0.5 and 5; values below 1.5 were considered indicative of low CH, values between 1.6 and 2 were considered indicative of intermediate CH; and values higher than 2 were considered indicative of high CH. All statistical analyses were carried out using the SPSS version 21 and *P* values < 0.05 were considered as statistically significant.

Studies have shown that FISH enables an estimation of CH within the tumor and allows the differentiation between CIN (aneuploid tumors with high CH) and stable aneuploidy (low CH)^29,36^. Therefore, we calculated the percentage of nuclei deviating from the modal centromeric signal for ­chromosomes 2, 3, 8, 11, 15, and 17 separately using established methods^29,31^. CIN and SDI are expressed as means ± SD.

## Results

### Definition of CIN levels and CH in control cell lines

**Figure 1:**
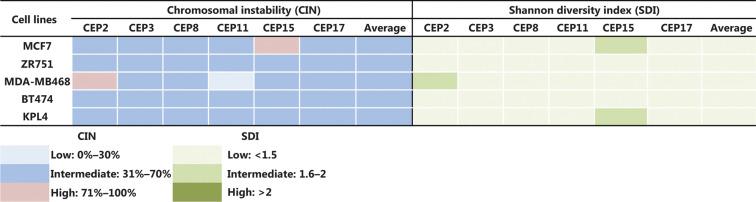
CIN and SDI in untreated BC cell lines. The level of CIN and the SDI (indicative of CH) in the five cell lines is color coded according to the legend at the bottom.

Some previous studies in BC patients classified high CIN as CIN ≥ 50%^24,34^, however, we considered CIN ≥ 50% as intermediate in this study for all cell lines, in order to more clearly show the variations in CIN levels after treatments. Therefore, we considered CIN as high when CIN > 70%, as indicated in the Materials and methods section. The CIN for the five BC cell lines analyzed in this study (MCF7, ZR751, MDA-MB468, BT474, and KPL4) ranged from 50% to 64% and the CH ranged from 1.18 to 1.5 (**Supplementary Table S2**). These cell lines were defined as having intermediate CIN and low CH (**Figure 1**). All untreated cell lines harbored the same level of CIN and CH at 24 h, 48 h, and 96 h. High CIN was associated with high CH, which can be observed mainly for chromosomes 2 and 15.

### Variation of CIN and CH in HER2- cells after treatments

#### MCF7 cells

**Figure 2:**
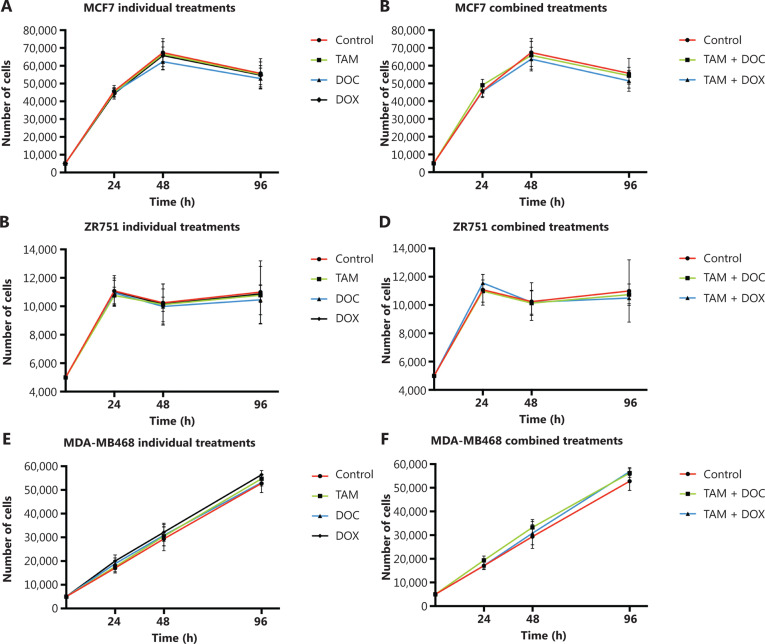
Effects of tamoxifen (TAM), docetaxel (DOC), doxorubicin (DOX), TAM+DOC and TAM+DOX treatments for 24 h, 48 h, and 96 h on cell proliferation in (A, B) MCF7 cells, (C, D) ZR751 cells and (E, F) MDA-MB468 cells. Error bars represent mean standard deviation of 24 replicates.

**Figure 3:**
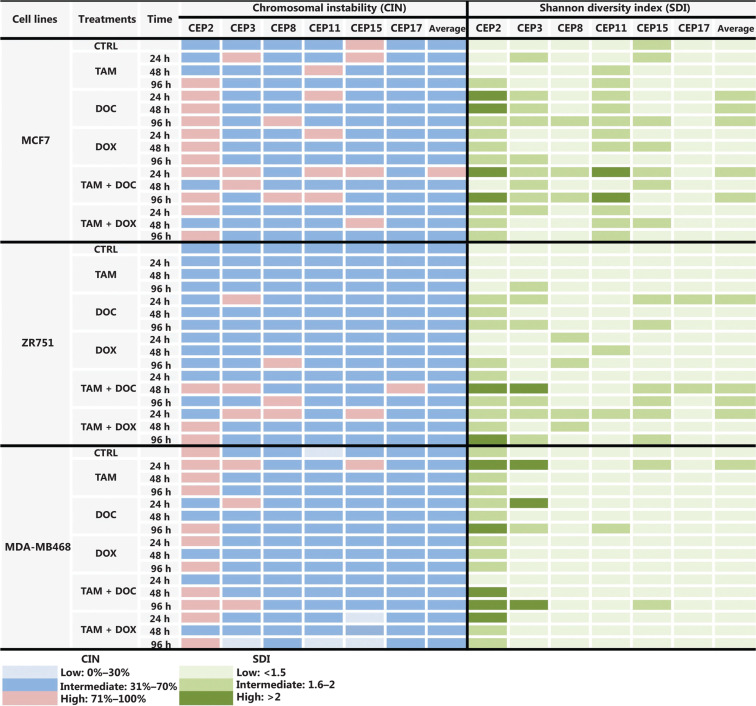
CIN and SDI (indicative of CH) in HER2- BC cells treated with tamoxifen (TAM), docetaxel (DOC), doxorubicin (DOX), TAM+DOC and TAM+DOX at various time points. The level of CIN and the SDI before and after treatments in ERa+/PR+/HER2− cells (MCF7 and ZR751) and ERa−/PR−/HER2− cells (MDA-MB468) is color coded according to the legend at the bottom.

**Figure 4:**
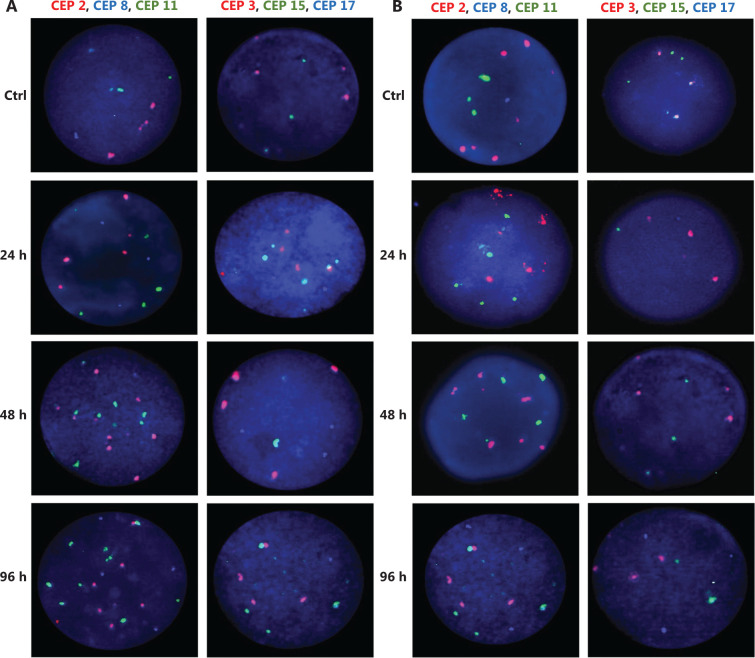
Representative FISH images of the MCF7 BC cells after (A) DOC treatment and (B) TAM+DOX treatment. Three-color FISH was performed on nuclei spreads for chromosomes 2, 8 and 11 and, chromosomes 3, 15 and 17 using centromeric probes (CEP) labeled with different spectrum colors: spectrum orange for CEP2 and CEP3; spectrum aqua for CEP8 and CEP17; and spectrum green for CEP11 and CEP15. Interphase nuclei at each treatment time point are indicated. Ctrl: Control, untreated cells.

In MCF cells (ERα+/PR+/HER2−), a greater reduction in cell proliferation was observed when individual and combined treatments did not increase the CIN above 68% (**Figures 2A, 2B, and 3, and Supplementary Table S2**). Although no statistically significant differences were observed (*P* ≤ 0.2, Student’s *t*-test), a trend towards reduced cell proliferation in response to all individual and combined treatments was evident (**Figures 2A and 2B**), as well as an increase in CIN from 64% in untreated cells to 68% after treatment (**Figure 3**). Among the monotherapies, DOC induced a greater reduction in cell proliferation (**Figure 2A**), and also both, an increase in CIN, which did not exceed 68% (**Figures 3 and 4**), and an increase in CH (from low to intermediate CH). These results show that cells with intermediate CIN respond better to treatment with taxanes (DOC) than with anthracyclines (DOX).

Both combined therapies, TAM+DOC and TAM+DOX, inhibited MCF7 cell proliferation, with a greater inhibition when TAM was combined with DOX (TAM+DOX) (**Figure 2B**). Notably, at 24 h of TAM+DOC, we observed significantly stimulated cell proliferation (*P* < 0.012, Student’s *t*-test) and increased CIN and CH (CIN = 72% and CH = 1.74) with respect to the untreated cells (CIN = 64% and CH = 1.5) (**Figure 3 and Supplementary Table S2**).

#### ZR751 cells

In ZR751 cells (ERα+/PR+/HER2−), a greater reduction in proliferation after individual or combined treatments was observed when the CIN increased, but did not exceed 67% (increased from 50% in untreated cells to 67% after treatments) (**Figure 3 and Supplementary Table S2**). Among the individual treatments, DOC induced a greater reduction in cell proliferation, and an increase in CIN, which did not exceed 67% (**Figure 2C and Figure 3**). Similar to results observed in MCF7 cells, in ZR751 cells, although no statistically significant differences were evidenced (*P* ≤ 0.3), a decrease in cell proliferation was observed in response to combined therapies (TAM+DOC and TAM+DOX), being identified a greater inhibition when TAM was combined with DOX (TAM + DOX) (**Figure 2D**). To highlight that in these cells, TAM+DOX treatment for 24 h stimulated cell proliferation and also increased CIN and CH (CIN = 70% and CH = 1.7) compared with the control (CIN = 50% and CH=1.2) (**Figure 3 and Supplementary Table S2**).

These results suggest that ERα+/PR+/HER2− tumor cells (MCF7 and ZR751), with intermediate CIN, respond better to treatment with taxanes (DOC) and to combined treatments with TAM+DOX compared with the treatment with anthracyclines and with TAM+DOC. Interestingly, the response is related to an increase in CIN no greater at 68%. In contrast, an increase in CIN equal to or greater than 70% was related to treatment resistance (**Supplementary Table S2**).

Notably, in all treatments applied and at all times (24 h, 48 h, and 96 h), a direct association between CIN and CH was observed, where at higher CIN also a higher CH was observed (**Supplementary Table S2**).

#### MDA-MB468 cells

In triple negative cells (ERα−/PR−/HER2−), all ­individual treatments stimulated cell proliferation (**Figure 2E**) and induced an increase in CIN from 50% in untreated cells to 67% after treatments (**Supplementary Table S2**). However, these cells after treatments presented a relatively stable CH (low CH).

**Figure 5:**
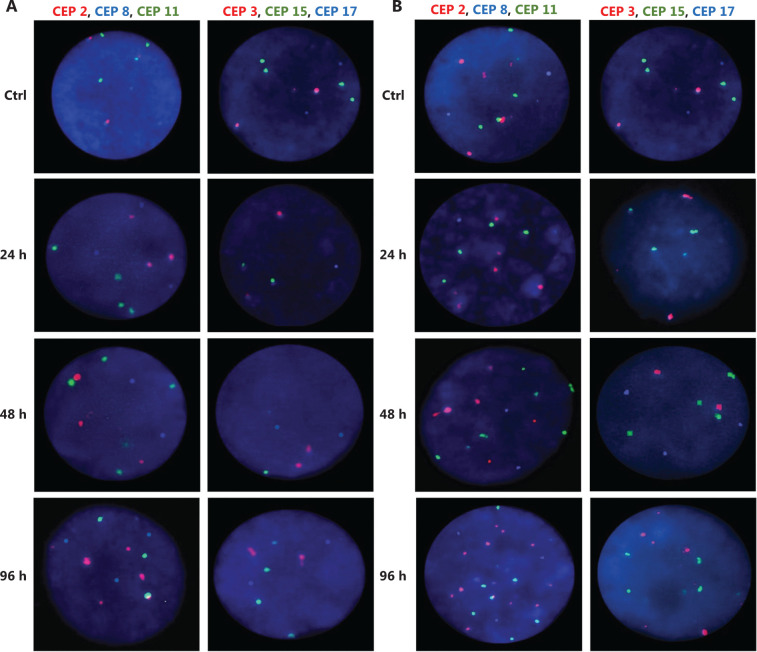
Representative FISH images of the MDA-MB468 BC cells after (A) TAM+DOC treatment and (B) DOX treatment. Three-color FISH was performed on nuclei spreads for chromosomes 2, 8 and 11 and, chromosomes 3, 15 and 17 using centromeric probes (CEP) labeled with different spectrum colors: spectrum orange for CEP2 and CEP3; spectrum aqua for CEP8 and CEP17; and spectrum green for CEP11 and CEP15. Interphase nuclei at each treatment time point are indicated. Ctrl: Control, untreated cells.

Among the individual treatments, DOX showed the poorest effects, as it not only significantly stimulated cell proliferation (*P* ≤ 0.01, Student’s *t*-test), but also increased CIN from 50% in untreated cells to 57% after treatment, and CH from 1.18 in untreated cells to 1.36 after treatment. Similar results were observed for the combined treatments, in which TAM+DOC and TAM+DOX treatments significantly stimulated cell proliferation (*P* ≤ 0.02) and increased CIN, which did not exceed 64% (**Figures 3 and 5**).

These results suggest that triple negative cells with intermediate CIN and low CH are resistant to chemotherapy (taxanes and anthracyclines), hormonal therapy and combined treatments (chemotherapy + hormonal treatment).

### Variation of CIN and CH after treatments in HER2+ cells

#### BT474 cells

**Figure 6:**
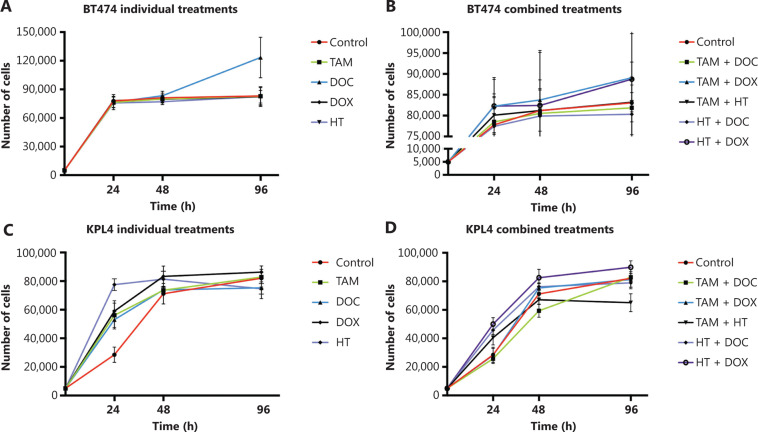
Effects of tamoxifen (TAM), docetaxel (DOC), doxorubicin (DOX), herceptin (HT), TAM+DOC, TAM+DOX, TAM+HT, HT+DOC and HT+DOX treatments for 24 h, 48 h, and 96 h on proliferation in (A, B) BT474 cells and (C, D) KPL4 cells. Error bars represent mean standard deviation of 24 replicates.

**Figure 7:**
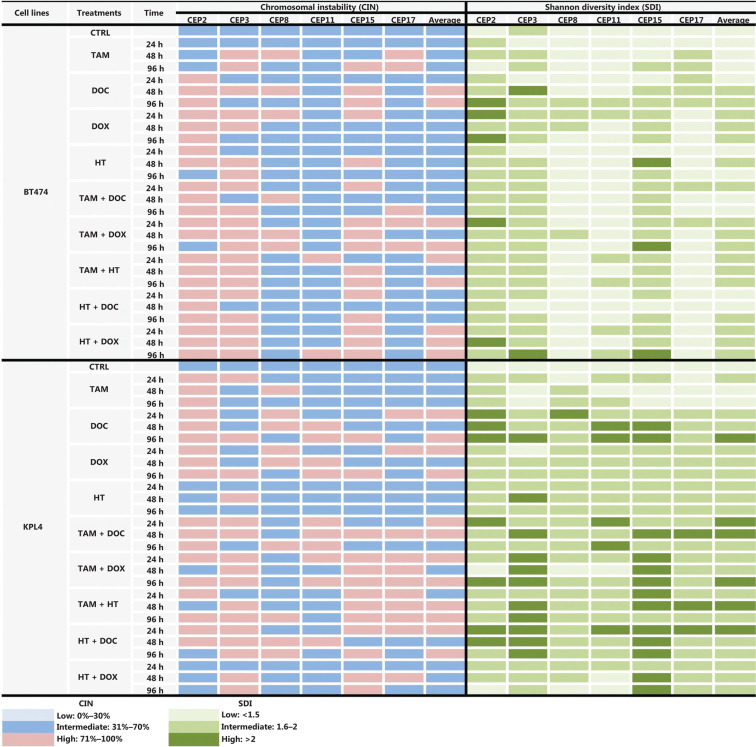
CIN and SDI (indicative of CH) induced by tamoxifen (TAM), docetaxel (DOC), doxorubicin (DOX), Herceptin (HT), TAM+DOC, TAM+DOX, TAM+HT, HT+DOC and HT+DOX in HER2+ BC cells at each treatment time point. The level of CIN and the SDI before and after treatments in ER+/PR+/HER2+ cells (BT474) and ER−/PR−/HER2+ cells (KPL4) is color coded according to the legend at the bottom.

In ERα+/PR+/HER2+ cells, a greater decrease in cell proliferation was observed when the treatments applied individually or in combination did not increase the CIN above 68% (**Figures 6 and 7, and Supplementary Table S2**). Among the individual treatments, HT was the treatment that showed the greatest inhibition of cell proliferation, also inducing an increase in CIN from 61% in untreated cells to 67% after treatment. In contrast, DOC significantly stimulated cell proliferation (*P* ≤ 0.001, **Figure 6A**) mainly at 96 h and induced an increase in CIN (72%) and CH (1.7) (**Figure 7 and Supplementary Table S2**). However, the combination of DOC and HT was superior to the use of chemotherapy with taxane alone, in which HT+DOC treatment inhibited cell proliferation (**Figure 6B**) and increased CIN to 65% compared with the untreated cells (61%). Similar results were observed when DOX was used as monotherapy, where it inhibited cell proliferation and increased CIN below 68%. While the administration of DOX, combined with TAM or HT, stimulated cell proliferation and increased CIN (above 69%) and CH (between 1.6 and 1.8).

Considering that chromosome 17 remained stable throughout the treatments and no major alterations were observed in the modal number (**Figure 7**), we could suggest that its stability is indicative of resistance to anthracyclines, in contrast to a previous report^37^, which indicated that tumor cells with CEP17 duplication (instability) may be associated with preferential sensitivity to ­anthracycline-based regimens.

#### KPL4 cells

In ERα−/PR−/HER2+ cells, all individual treatments showed the poorest effects, as it not only significantly stimulated cell proliferation (*P* ≤ 0.0037, **Figure 6C**), but also increased CIN from 50% in untreated cells to 68% after treatments (**Figure 7 and Supplementary Table S2**). Contrary to what was observed in ERα+/PR+/HER2−, ERα+/PR+/HER2+ and ERα−/PR−/HER2− cells, in KPL4 cells (ERα−/PR−/HER2+), a greater decrease in cell proliferation was observed when the treatments administered in combination increased the CIN above 70% (**Figures 6D and 7, and Supplementary Table S2**). Only the combined treatments between TAM with DOC, DOX, or HT, showed reduction in cell proliferation, with a greater reduction being observed when TAM was combined with taxanes (*P* ≤ 0.0005, **Figure 6D**). The administration of TAM+DOC increased both CIN, from 50% in the untreated cells to 74% after treatment (**Supplementary Figure S1**) and CH, from 1.31 in the untreated cells to 2 after treatment (**Supplementary Table S2**). Whereas HT administered as a single agent or in combination with chemotherapy (­anthracyclines or taxanes) induced an increase in both cell proliferation (*P* ≤ 0.0003, **Figure 6D**) and CIN of 50% in untreated cells to 68% after treatment.

## Discussion

BC is a heterogeneous disease in which each tumor shows individual characteristics. This has led to the search for new markers to improve not only patient diagnosis but also to obtain a better response to therapy and improve prognosis. Currently, strategies for the treatment of BC depend on the tumor subtype, in which therapies are selected based on specific markers. For example, for tumors with positive hormone receptors (ERα and PR) (luminal A and luminal B), endocrine therapy (TAM) is applied^38,39^, with some patients also requiring chemotherapy. In the case of HER2+ tumors (Luminal B and HER2+), the treatment consists of monoclonal antibodies that recognize the extracellular domain of HER2 (HT), as well as inhibitors of the tyrosine kinase domain of the HER2 receptor (such as lapatinib)^40^, in addition to endocrine therapy (TAM) if the hormone receptors are also positive. For triple negative BC, only chemotherapy is applied. Chemotherapy is the only systemic therapy with proven efficacy in triple negative BC and an important complement of endocrine therapy or therapy directed to HER2 in patients with BC positive for hormone receptors (ER and PR)^41^. However, it is important to highlight that although the ERα positivity is a well-established predictor of response to TAM and the patients negative for ERα are considered non-responders, it has been reported that around 5%–10% of patients with ERα− tumors benefit from adjuvant treatment with TAM^42–46^.

In this study, we observed that intermediate CIN is linked to drug sensitivity according to three characteristics, including ER and HER2 status, the pre-existing level of CIN of the tumor cells, and the CIN induced by the treatments. In addition, all therapies used in our study (monotherapy or combined therapy) promoted CIN themselves, which influenced the response to therapy. We found that ERα+/PR+/HER2− cells with intermediate CIN were sensitive to treatment with taxanes (DOC) and anthracyclines (DOX), while ERα−/PR−/HER2− and ERα+/PR+/HER2+ cells with intermediate CIN were resistant to these drugs, mainly when anthracyclines were used in combination with TAM. These results are consistent with previous studies that indicate that while ERα+/PR+/HER2− and ERα+/PR+/HER2+ tumoral cells showed the poorest prognosis with both shorter time to progression and overall survival, ERα+/PR+/HER2− tumoral cells had a considerably better prognosis^47–49^. In addition, HER2+ cells are generally more proliferative than HER2− cells. Increased cell proliferation could lead to the expansion of chromosomal alterations and therefore a general increase in CIN and intratumor CH. Both CIN and CH increased cellular diversity allowing tumors to adapt to various micro-environmental selection pressure^50–52^. This adaptive ability may also lead to drug resistance^53–55^. While in triple negative tumoral cells (ERα−/PR−/HER2−) has been reported that resistance may be caused by preexisting (inherent) genetic mutations that cause a decrease in the responsiveness of cancer cells to both chemotherapy and target drugs^56^. The above finding as well as our results, suggests a difference in the mechanisms of therapy response according to ERα and HER2 status and highlights that these BC cell lines are distinct biological entities.

In ERα+/PR+/HER2+ cells (BT474), DOC stimulated cell proliferation and increased CIN, which exceeds 70%, while in ERα+/PR+/HER2− cells (MCF7 and ZR751 cells), DOC inhibited cell proliferation and increased CIN at 68%. Thus, in ERα+/PR+/HER2− cells, the induction of greater CIN above the pre-existing CIN (before treatment) but below 70% (after treatment) indicates sensitivity to therapy (inhibition of cell proliferation). However, in ERα+/PR+/HER2- cells, if the CIN threshold is exceeded by 70% and CH also increases, cells develop resistance to therapy. The increased rate of CIN and CH may inadvertently create a more aggressive tumor with an enhanced potential to become drug resistant.

DOC is generally recognized as one of the most effective drugs available for the treatment of metastatic BC^57^ and is studied in patients who do not respond or develop resistance to chemotherapy with anthracyclines (DOX)^58,59^. However, although we observed that DOC was more effective than DOX when it was administered to ERα+/PR+/HER2− tumor cells (MCF7 and ZR751) with intermediate CIN, it was not effective when administered to ERα+/PR+/HER2+ cells (BT474), where DOC induced an increase in cell proliferation compared to the control.

In fact, in ERα+/PR+/HER2+ cells, intermediate CIN (61%) predicts sensitivity to treatment with TAM, DOX, HT, DOC+TAM and DOC+HT and resistance to DOC, TAM+HT, TAM+DOX and HT+DOX. Our results are consistent with those reported in breast and colorectal tumors, in which taxanes (DOC) inhibited cell proliferation when applied to tumor cells with low CIN, but induced resistance when applied to tumor cells with intermediate or high CIN^2^. Furthermore, the resistance to TAM+DOX, TAM+HT and HT+DOX observed in these cells (ERα+/PR+/HER2+ cells), with intermediate CIN and intermediate CH, could be caused by the presence of sub clonal populations with different levels of CIN, and therefore with varied responses to treatments. For example, in a subpopulation of tumor cells with a pre-­existing level of “intermediate or high CIN”, drugs that induce CIN could increase the level of CIN beyond a critical threshold thereby causing cytotoxicity, while the viability of a subpopulation of tumor cells with “low CIN” may not be compromised. Together these results suggest the effectiveness of the combined use of taxanes and therapies directed against HER2 in ERα+/PR+/HER2+ cells, since these drugs may target karyotypically distinct subpopulations within the same tumor (intratumoral CH)^60^.

In contrast to the results in BT474 cells (ERα+/PR+/HER2+), triple negative cells ERα−/PR−/HER2− with complex karyotypes (intermediate CIN = 50%) and low CH (stable CIN) were resistant to all treatments used. Although all treatments increased CIN, this was possibly not enough to exceed the tolerance threshold of the cells, leading to cell survival. Because we did not observe an inhibition in cell proliferation with any of the treatments, it is not possible for us to postulate a CIN threshold below or above which the treatments can induce cell survival or lethality. These results might suggest that many cancer cells demonstrate adaptation to CIN*,* suggesting that maintaining CIN and CH may favor cancer cell viability and survival. Thus, and as indicated by Thompson et al.^61^, it is possible that triple negative cancer cells with positive CIN may exhibit intrinsic resistance to drugs that seek to increase CIN through a similar mechanism. Such intrinsic resistance can be caused by pre-existing (inherent) genetic mutations that result in decreased responsiveness of cancer cells to both chemotherapy and targeted drugs or by heterogeneity of tumors in which pre-existed insensitive subpopulations will be selected upon drug treatment, thus leading to resistance to therapeutic treatments^62^.

The results observed in KPL4 cells (ERα−/PR−/HER2+), suggest that HER2 overexpression may influence the response to taxanes, where TAM significantly increase the antitumor activity of DOC*,* also avoiding an increase in CIN and CH. The results observed in ERα−/PR−/HER2+ cells are consistent with previous studies that have shown that HER2 overexpression predicts a worse response to hormonal therapy with TAM in BC^63,64^. Although ERα−/PR−/HER2+ cells are negative for ERα, the best results in terms of reduction in cell proliferation were observed when combined treatments between TAM with DOC, DOX, or HT were administered to the cells. These results are not surprising as it has been reported that although ERα− patients are considered non-responders, between 5% and 10% of them benefit from TAM therapy^43–45^. Such a response could be due to a member of the 7-transmembrane G protein-coupled receptor family, GPR30, an estrogen transmembrane receptor involved in mediating both rapid and transcriptional events in response to estrogen^65–67^. Of note, similar to recent reports^68^, we observe that the combination of HER2-targeted therapy (HT) with endocrine therapy (TAM) was superior to endocrine therapy alone for ER−/PR−/HER2+ cells, where a reduction in cell proliferation and an increase in CIN greater than 70% was observed. These findings not only suggest a possible benefit of the use of these combinations as a treatment option, but also of the role of CIN in the development of cytotoxicity. Contrary to what was observed in MCF7, ZR751, MDA-MB468, and BT474 cells, in ERα−/PR−/HER2+ cells (KPL4) cells a high CIN is related to a high sensitivity to combined treatments.

In HER2+ cells (BT474 and KPL4), a greater benefit in terms of reduction of cell proliferation was observed when the combined treatment between trastuzumab (HT) and chemotherapy (DOC) was administered, compared to that observed when chemotherapy was administered alone. Such a benefit was also reported in an open-label randomized multicenter phase II trial (M77001), where the efficacy and safety of first-line HT with DOC compared with DOC alone in patients with HER2+ was observed^69^. Considering this, we suggest that in cells HER2+ (BT474 and KPL4), HER2 overexpression is a predictor of resistance to taxanes.

Considering that in KPL4 cells a CIN greater than 69% was related to sensitivity or response to therapy, it could be suggested that an increase in the dose of the treatments (applied in monotherapy) could be necessary to increase CIN and to induce cytotoxicity. In fact, some studies have reported that HER2 overexpression may be a useful marker for identifying patients who are most likely to benefit from high doses of adjuvant doxorubicin-based chemotherapy. Furthermore, previous studies demonstrated that in ERα− BC, extreme CIN is associated with improved clinical outcome, which is consistent with a negative impact of CIN on tumor fitness and growth^18^. The results observed in ERα−cells (MDA-MB468 and KPL4) with intermediate CIN, suggest the need to modify the dose of the drugs to generate a higher CIN and, therefore, cellular toxicity. Therefore, defining the maximum dose at which these treatments could induce a higher CIN without side effects is required. Recently, new treatment strategies of “on and off” or “high dose followed by low dose” have been used, which result in longer survival and delayed drug resistance. This may be because intermittent or adaptive dosing may interrupt the growth of drug-dependent resistant cells and allow the competition of sensitive and resistant cells^70^.

Related to resistance due to the tumor subtype, Davis and colleagues^71^ reported that the MCF7 cell line (ERα+/PR+/HER2−), representative of the luminal A tumor subtype, is sensitive to DOX, while the MDA-MB231 cell line (ERα−/PR−/HER2−), representative of the triple negative tumor subtype, is resistant to DOX. This report is consistent with our results, as we observed that the MCF7 and ZR751 cell lines, both ERα+/PR+/HER2− and representative of the luminal A BC subtype, are sensitive to DOX treatment, while the MDA-MB468 (ERα−/PR−/HER2−) and KPL4 cell lines (ERα−/PR−/HER2+), representatives of triple negative and HER2+ BC subtype, respectively, did not respond to this drug. Moreover, the addition of DOX to MCF7 and ZR751 increased the CIN but did not exceed 67%, whereas in the KPL4 cells, DOX increased the CIN but did not exceed 69%.

**Figure 8:**
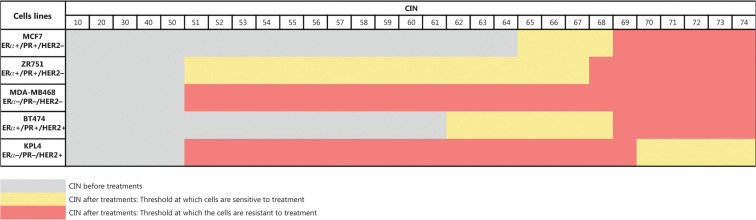
CIN threshold at which cells are sensitive or resistant to treatments.

Our results suggest that indeed a CIN threshold exists, which when exceeded could induce cell cytotoxicity or cell survival. For instance, in ERα+/PR+/HER2− cells (MCF7 and ZR751) and ERα+/PR+/HER2+ cells (BT474) with intermediate CIN, the therapy (chemotherapy, hormonal therapy, or combined therapies) induced an increase in CIN greater than 70% and a higher CH, which correlated to cell survival. While in ERα−/PR−/HER2+ cells (KPL4) with intermediate CIN, the monotherapy and combined therapy induced an increase in CIN greater than 70% and a higher CH, which correlated to cell cytotoxicity (**Figure 8**). This behavior suggests a difference in mechanisms of therapy response according to ERα and HER2 status, and highlights that these BC cell lines are distinct biological entities, as previously indicated.

Considering that high CH is the likely result of aneuploidy originating from CIN generating multiple unrelated clones, we speculate that both the baseline CH and CIN and those generated after drug exposure are sufficiently diverse to confer proliferative advantages and therefore resistance. Opposite results were observed in triple negative cells (ERα−/PR−/HER2−), in which monotherapy and combined treatments induced an increase in proliferation. These cells showed an increase in CIN that did not exceed 68% and a relative stability (low CH).

Consistent with a close relationship of CIN with CH in BC, there was a high association between CIN and SDI for all chromosomes and for all cell lines studied before and after treatments (**Figures 3 and 7 and Supplementary Table S2**). These data suggest that cancer cells with high CIN (CIN > 71%) have the highest SDI and thus the most extreme chromosomal numerical heterogeneity (**Figures 3 and 7, and Supplementary Table S2**). These results are concordant with the “unstable aneuploidy” definition provided by Lingle and colleagues in an analysis of 20 breast tumors^29^.

The exploratory analysis of the individual chromosomes in all cell lines analyzed (MCF7, ZR751, MDA-MB468, BT474, and KPL4) highlighted chromosome (Chr) 2, Chr8, and Chr17 as candidate markers of the therapy response. Chr2 showed an unstable behavior (high CIN and high CH) after individual and combined treatments in all cell lines, in comparison with Chr8 in BT474 cells and Chr17 in MCF7, ZR751, and MDA-MB468 cells, which showed stable behavior (intermediate CIN and low CH) after treatments. In ERα+/PR+/HER2− cells, Chr17 stability is indicative of sensitivity to treatment with taxanes (DOC) and combined treatments of anthracyclines (DOX) with TAM, while in ERα−/PR−/HER2− cells, Chr17 stability is indicative of resistance to chemotherapy (DOC, DOX), hormonal therapy (TAM) and combined treatments of taxanes and anthracyclines with TAM. In ERα+/PR+/HER2+ cells, Chr8 stability is indicative of sensitivity to treatment with TAM, DOX, HT and combined treatments of taxanes (DOC) with TAM and with HT.

## Conclusions

Our results suggest that intermediate CIN in BC tumors is of prognostic value and may be able to predict response to chemotherapy, hormonal therapy, or combined therapy. We also suggested the existence of a CIN threshold that when it is exceeded can generate resistance or sensibility to treatments, and such a CIN threshold is dependent on ERα and HER status. Our data require prospective validation in larger patient cohorts with defined tumor stage and treatment history.

Tumors classified due to their underlying CIN may provide a more thorough understanding of alterations at the molecular level and potentially lead to new drug targets. A greater understanding of the role of chemotherapy, hormonal therapy, or combined therapy in the induction of CIN and CH in BC together with prospective studies of CIN in patients with BC could contribute to the optimization of existing therapeutic regimens. Our results emphasize the importance of determining CIN level and CH in BC tumors to direct the most effective therapeutic strategy for an individual BC patient.

## References

[1] Pikor L, Thu K, Vucic E, Lam W (2013). The detection and implication of genome instability in cancer. Cancer Metastasis Rev.

[2] Burrell RA, Juul N, Johnston SR, Reis-Filho JS, Szallasi Z, Swanton C (2010). Targeting chromosomal instability and tumour heterogeneity in HER2-positive breast cancer. J Cell Biochem.

[3] Tanaka K, Hirota T (2016). Chromosomal instability: a common feature and a therapeutic target of cancer. Biochim Biophys Acta.

[4] Geigl JB, Obenauf AC, Schwarzbraun T, Speicher MR (2008). Defining ‘chromosomal instability’. Trends Genet.

[5] Gagos S, Irminger-Finger I (2005). Chromosome instability in neoplasia: chaotic roots to continuous growth. Int J Biochem Cell Biol.

[6] Thompson SL, Compton DA (2011). Chromosomes and cancer cells. Chromosome Res.

[7] Heng HH, Bremer SW, Stevens JB, Horne SD, Liu G, Abdallah BY (2013). Chromosomal instability (CIN): what it is and why it is crucial to cancer evolution. Cancer Metastasis Rev.

[8] Chandrakasan S, Ye CJ, Chitlur M, Mohamed AN, Rabah R, Konski A (2011). Malignant fibrous histiocytoma two years after autologous stem cell transplant for Hodgkin lymphoma: evidence for genomic instability. Pediatr Blood Cancer.

[9] Isola JJ, Kallioniemi OP, Chu LW, Fuqua SA, Hilsenbeck SG, Osborne CK (1995). Genetic aberrations detected by comparative genomic hybridization predict outcome in node-negative breast cancer. Am J Pathol.

[10] Ried T, Just KE, Holtgreve-Grez H, du Manoir S, Speicher MR, Schrock E (1995). Comparative genomic hybridization of formalin-fixed, paraffin-embedded breast tumors reveals different patterns of chromosomal gains and losses in fibroadenomas and diploid and aneuploid carcinomas. Cancer Res.

[11] Persson K, Pandis N, Mertens F, Borg A, Baldetorp B, Killander D (1999). Chromosomal aberrations in breast cancer: a comparison between cytogenetics and comparative genomic hybridization. Genes Chromosomes Cancer.

[12] Heim S, Mitelman F (2015). Cancer cytogenetics: Fourth edition.

[13] Dayal J, Albergant L, Newman T, South A (2015). Quantitation of multiclonality in control and drug-treated tumour populations using high-throughput analysis of karyotypic heterogeneity. Converg. Sci Phys Oncol.

[14] Fedorenko IV, Wargo JA, Flaherty KT, Messina JL, Smalley KS (2015). BRAF inhibition generates a host-tumor niche that mediates therapeutic escape. J Invest Dermatol.

[15] Bartlett JM, Munro AF, Dunn JA, McConkey C, Jordan S, Twelves CJ (2010). Predictive markers of anthracycline benefit: a prospectively planned analysis of the UK National Epirubicin Adjuvant Trial (NEAT/BR9601). Lancet Oncol.

[16] O’Malley FP, Chia S, Tu D, Shepherd LE, Levine MN, Bramwell VH (2009). Topoisomerase II alpha and responsiveness of breast cancer to adjuvant chemotherapy. J Natl Cancer Inst.

[17] Birkbak NJ, Eklund AC, Li Q, McClelland SE, Endesfelder D, Tan P (2011). Paradoxical relationship between chromosomal instability and survival outcome in cancer. Cancer Res.

[18] Jamal-Hanjani M, A’Hern R, Birkbak NJ, Gorman P, Gronroos E, Ngang S (2015). Extreme chromosomal instability forecasts improved outcome in ER-negative breast cancer: a prospective validation cohort study from the tact trial. Ann Oncol.

[19] Roylance R, Endesfelder D, Gorman P, Burrell RA, Sander J, Tomlinson I (2011). Relationship of extreme chromosomal instability with long-term survival in a retrospective analysis of primary breast cancer. Cancer Epidemiol Biomarkers Prev.

[20] Sapino A, Pietribiasi F, Bussolati G, Marchisio PC (1986). Estrogen- and tamoxifen-induced rearrangement of cytoskeletal and adhesion structures in breast cancer MCF-7 cells. Cancer Res.

[21] Hartmann K, Becker-Putsche M, Bocklitz T, Pachmann K, Niendorf A, Rosch P (2012). A study of docetaxel-induced effects in MCF-7 cells by means of Raman microspectroscopy. Anal Bioanal Chem.

[22] Wang S, Konorev EA, Kotamraju S, Joseph J, Kalivendi S, Kalyanaraman B (2004). Doxorubicin induces apoptosis in normal and tumor cells via distinctly different mechanisms. Intermediacy of H(2)O(2)- and p53-dependent pathways. J Biol Chem.

[23] Ginestier C, Adelaide J, Goncalves A, Repellini L, Sircoulomb F, Letessier A (2007). ERBB2 phosphorylation and trastuzumab sensitivity of breast cancer cell lines. Oncogene.

[24] Munro AF, Twelves C, Thomas JS, Cameron DA, Bartlett JM (2012). Chromosome instability and benefit from adjuvant anthracyclines in breast cancer. Br J Cancer.

[25] Cisyk AL, Penner-Goeke S, Lichtensztejn Z, Nugent Z, Wightman RH, Singh H (2015). Characterizing the prevalence of chromosome instability in interval colorectal cancer. Neoplasia.

[26] Choi CM, Seo KW, Jang SJ, Oh YM, Shim TS, Kim WS (2009). Chromosomal instability is a risk factor for poor prognosis of adenocarcinoma of the lung: fluorescence *in situ* hybridization analysis of paraffin-embedded tissue from Korean patients. Lung Cancer.

[27] Nakamura H, Saji H, Idiris A, Kawasaki N, Hosaka M, Ogata A (2003). Chromosomal instability detected by fluorescence *in situ* hybridization in surgical specimens of non-small cell lung cancer is associated with poor survival. Clin Cancer Res.

[28] Rondon-Lagos M, Verdun Di Cantogno L, Marchio C, Rangel N, Payan-Gomez C, Gugliotta P (2014). Differences and homologies of chromosomal alterations within and between breast cancer cell lines: a clustering analysis. Mol Cytogenet.

[29] Lingle WL, Barrett SL, Negron VC, D’Assoro AB, Boeneman K, Liu W (2002). Centrosome amplification drives chromosomal instability in breast tumor development. Proc Natl Acad Sci U S A.

[30] Chin K, de Solorzano CO, Knowles D, Jones A, Chou W, Rodriguez EG (2004). *In situ* analyses of genome instability in breast cancer. Nat Genet.

[31] Fiegl M, Kaufmann H, Zojer N, Schuster R, Wiener H, Mullauer L (2000). Malignant cell detection by fluorescence *in situ* hybridization (FISH) in effusions from patients with carcinoma. Hum Pathol.

[32] Takami S, Kawasome C, Kinoshita M, Koyama H, Noguchi S (2001). Chromosomal instability detected by fluorescence in situ hybridization in Japanese breast cancer patients. Clin Chim Acta.

[33] Farabegoli F, Santini D, Ceccarelli C, Taffurelli M, Marrano D, Baldini N (2001). Clone heterogeneity in diploid and aneuploid breast carcinomas as detected by FISH. Cytometry.

[34] Lengauer C, Kinzler KW, Vogelstein B (1997). Genetic instability in colorectal cancers. Nature.

[35] Maley CC, Galipeau PC, Finley JC, Wongsurawat VJ, Li X, Sanchez CA (2006). Genetic clonal diversity predicts progression to esophageal adenocarcinoma. Nat Genet.

[36] Chin K, DeVries S, Fridlyand J, Spellman PT, Roydasgupta R, Kuo WL (2006). Genomic and transcriptional aberrations linked to breast cancer pathophysiologies. Cancer Cell.

[37] Burrell RA, McClelland SE, Endesfelder D, Groth P, Weller MC, Shaikh N (2013). Replication stress links structural and numerical cancer chromosomal instability. Nature.

[38] Parisot JP, Hu XF, DeLuise M, Zalcberg JR (1999). Altered expression of the IGF-1 receptor in a tamoxifen-resistant human breast cancer cell line. Br J Cancer.

[39] Berry DA, Muss HB, Thor AD, Dressler L, Liu ET, Broadwater G (2000). HER-2/neu and p53 expression versus tamoxifen resistance in estrogen receptor-positive, node-positive breast cancer. J Clin Oncol.

[40] Tai W, Mahato R, Cheng K (2010). The role of HER2 in cancer therapy and targeted drug delivery. J Control Release.

[41] Waks AG, Winer EP (2019). Breast cancer treatment: a review. J Am Med Assoc.

[42] McGuire WL (1975). Current status of estrogen receptors in human breast cancer. Cancer.

[43] Early Breast Cancer Trialists’ Collaborative Group (1992). Systemic treatment of early breast cancer by hormonal, cytotoxic, or immune therapy. 133 randomised trials involving 31,000 recurrences and 24,000 deaths among 75,000 women. Lancet.

[44] Early Breast Cancer Trialists’ Collaborative Group (1998). Tamoxifen for early breast cancer: an overview of the randomised trials. Lancet.

[45] Davies C, Godwin J, Gray R, Clarke M, Cutter D, Early Breast Cancer Trialists’ Collaborative Group (2011). Relevance of breast cancer hormone receptors and other factors to the efficacy of adjuvant tamoxifen: patient-level meta-analysis of randomised trials. Lancet.

[46] Gruvberger-Saal SK, Bendahl PO, Saal LH, Laakso M, Hegardt C, Eden P (2007). Estrogen receptor beta expression is associated with tamoxifen response in ERalpha-negative breast carcinoma. Clin Cancer Res.

[47] Perou CM, Sorlie T, Eisen MB, van de Rijn M, Jeffrey SS, Rees CA (2000). Molecular portraits of human breast tumours. Nature.

[48] Sorlie T, Perou CM, Tibshirani R, Aas T, Geisler S, Johnsen H (2001). Gene expression patterns of breast carcinomas distinguish tumor subclasses with clinical implications. Proc Natl Acad Sci U S A.

[49] Sotiriou C, Neo SY, McShane LM, Korn EL, Long PM, Jazaeri A (2003). Breast cancer classification and prognosis based on gene expression profiles from a population-based study. Proc Natl Acad Sci U S A.

[50] Walther A, Houlston R, Tomlinson I (2008). Association between chromosomal instability and prognosis in colorectal cancer: a meta-analysis. Gut.

[51] Kronenwett U, Ploner A, Zetterberg A, Bergh J, Hall P, Auer G (2006). Genomic instability and prognosis in breast carcinomas. Cancer Epidemiol Biomarkers Prev.

[52] Gerlinger M, Swanton C (2010). How Darwinian models inform therapeutic failure initiated by clonal heterogeneity in cancer medicine. Br J Cancer.

[53] Swanton C, Nicke B, Schuett M, Eklund AC, Ng C, Li Q (2009). Chromosomal instability determines taxane response. Proc Natl Acad Sci U S A.

[54] Duesberg P, Stindl R, Hehlmann R (2000). Explaining the high mutation rates of cancer cells to drug and multidrug resistance by chromosome reassortments that are catalyzed by aneuploidy. Proc Natl Acad Sci U S A.

[55] Li R, Hehlman R, Sachs R, Duesberg P (2005). Chromosomal alterations cause the high rates and wide ranges of drug resistance in cancer cells. Cancer Genet Cytogenet.

[56] Wang Xuan ZH, Chen X (2019). Drug resistance and combating drug resistance in cancer. Cancer Drug Resist.

[57] Crown J (1999). A review of the efficacy and safety of docetaxel as monotherapy in metastatic breast cancer. Semin Oncol.

[58] Crown J (2001). Docetaxel: overview of an active drug for breast cancer. Oncologist.

[59] Verweij J, Clavel M, Chevalier B (1994). Paclitaxel (Taxol) and docetaxel (Taxotere): not simply two of a kind. Ann Oncol.

[60] Pack SD, Alper OM, Stromberg K, Augustus M, Ozdemirli M, Miermont AM (2004). Simultaneous suppression of epidermal growth factor receptor and c-erbB-2 reverses aneuploidy and malignant phenotype of a human ovarian carcinoma cell line. Cancer Res.

[61] Thompson LL, Jeusset LM, Lepage CC, McManus KJ (2017). Evolving therapeutic strategies to exploit chromosome instability in cancer. Cancers (Basel).

[62] Kelderman S, Schumacher TN, Haanen JB (2014). Acquired and intrinsic resistance in cancer immunotherapy. Mol Oncol.

[63] Wright C, Nicholson S, Angus B, Sainsbury JR, Farndon J, Cairns J (1992). Relationship between c-erbB-2 protein product expression and response to endocrine therapy in advanced breast cancer. Br J Cancer.

[64] Carlomagno C, Perrone F, Gallo C, De Laurentiis M, Lauria R, Morabito A (1996). c-erb B2 overexpression decreases the benefit of adjuvant tamoxifen in early-stage breast cancer without axillary lymph node metastases. J Clin Oncol.

[65] Thomas P, Pang Y, Filardo EJ, Dong J (2005). Identity of an estrogen membrane receptor coupled to a G protein in human breast cancer cells. Endocrinology.

[66] Chen JQ, Russo J (2009). ERalpha-negative and triple negative breast cancer: molecular features and potential therapeutic approaches. Biochim Biophys Acta.

[67] Cheng SB, Graeber CT, Quinn JA, Filardo EJ (2011). Retrograde transport of the transmembrane estrogen receptor, G-protein-coupled-receptor-30 (GPR30/GPER) from the plasma membrane towards the nucleus. Steroids.

[68] Ruta Rao MC (2019). HER2-positive breast cancer.

[69] Marty M, Cognetti F, Maraninchi D, Snyder R, Mauriac L, Tubiana-Hulin M (2005). Randomized phase II trial of the efficacy and safety of trastuzumab combined with docetaxel in patients with human epidermal growth factor receptor 2-positive metastatic breast cancer administered as first-line treatment: the M77001 Study Group. J Clin Oncol.

[70] Kaiser J (2017). When less is more. Science.

[71] Davis T, van Niekerk G, Peres J, Prince S, Loos B, Engelbrecht AM (2018). Doxorubicin resistance in breast cancer: a novel role for the human protein AHNAK. Biochem Pharmacol.

