# Supplementary material for: Role of chromosomal instability and clonal heterogeneity in the therapy response of breast cancer cell lines
Source: Cancer Biol Med. 2020 Dec 15;17(4):970–85. doi: 10.20892/j.issn.2095-3941.2020.0028 (PMC7721098; doi:10.20892/j.issn.2095-3941.2020.0028)
Supplement: Supplementary file 1 [file cbm-17-970-s001.pdf]

Supplementary materials

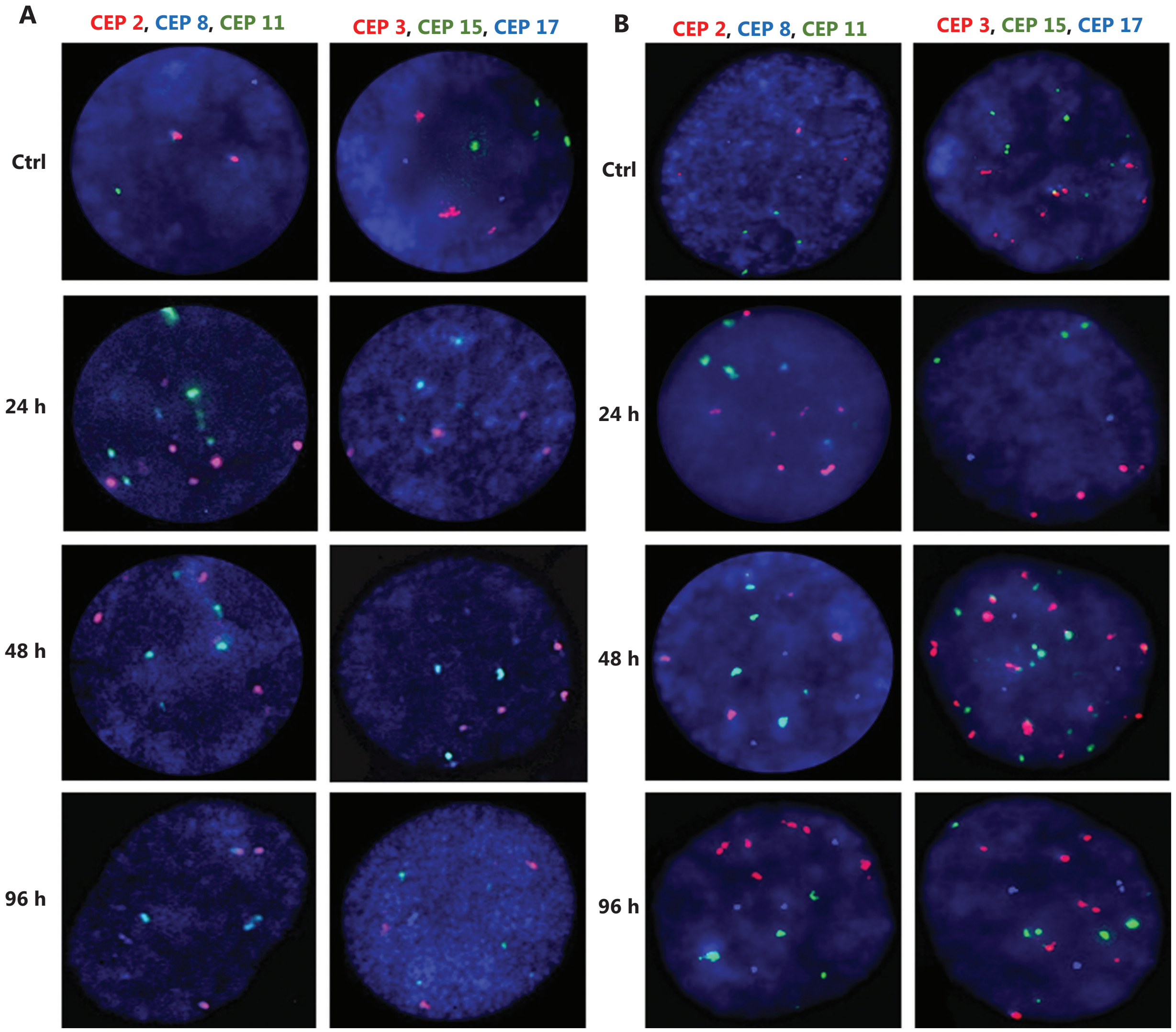

Figure S1 Representative FISH images of the KPL4 BC cells after (A) DOC treatment and (B) TAM+DOC treatment. Three-color FISH was performed on nuclei spreads for chromosomes 2, 8, and 11 and, chromosomes 3, 15, and 17 using centromeric probes (CEP) labeled with different spectrum colors, spectrum orange for CEP2 and CEP3; spectrum aqua for CEP8 and CEP17; and spectrum green for CEP11 and CEP15. Interphase nuclei at each treatment time point are indicated. Ctrl, control, untreated cells.

Table S1 BC cell lines and treatment strategies

Cell line	Receptor status	Individual treatments				Combined treatments				
		TAM	DOC	DOX	HT	TAM+DOC	TAM+DOX	TAM+HT	HT+DOC	HT+DOX
MCF7	ER+/PR+/HER2−	X	X	X		X	X			
ZR75-1	ER+/PR+/HER2−	X	X	X		X	X			
MDA-MB468	ER−/PR−/HER2−	X	X	X		X	X			
BT474	ER+/PR+/HER2+	X	X	X	X	X	X	X	X	X
KPL4	ER−/PR−/HER2+	X	X	X	X	X	X	X	X	X

TAM, tamoxifen; DOC, docetaxel; DOX, doxorubicin; HT, herceptin.

Table S2 CIN and SDI for all cell lines before and after treatments at 24 h, 48 h and 96 h

Cell line	Treatments	Time	CIN						Mean CIN	SD	SDI						Mean SDI	SD
			CEP2	CEP 3	CEP8	CEP 11	CEP 15	CEP17			CEP2	CEP 3	CEP8	CEP 11	CEP 15	CEP17		
MCF7 ER+/PR+/HER2−	CTRL		53	68	65	62	74	63	64	4.8	1.38	1.53	1.56	1.43	1.63	1.48	1.5	007
	TAM	24 h	60	75	52	62	72	67	65	6.6	1.49	1.68	1.12	1.22	1.6	1.38	1.41	1.18
		48 h	56	52	56	70	62	67	61	5.8	1.3	1.29	1.08	1.53	1.5	1.5	1.36	1.14
		96 h	74	62	65	68	50	53	62	7	1.84	1.35	1.45	1.55	1.14	1.18	1.41	0.2
	DOC	24 h	79	57	64	73	65	60	66	6.4	2;15	1.64	1.54	1.93	1.54	1.29	1.68	0.24
		48 h	79	67	70	62	66	62	68	4.5	202	1.67	1.54	1.8	1.54	1.3	1.64	0.19
		96 h	76	70	71	65	68	59	68	4.1	1.81	1.75	1.6	1.68	1.66	1.23	1.62	0.14
	DOX	24 h	75	63	60	74	68	62	67	5.3	1.88	1.36	1.4	1.77	1.58	1.25	1.54	0.2
		48 h	75	64	60	58	66	63	64	4.1	1.84	1.47	1.33	1.6	1.6	1.36	1.53	0.15
		96 h	72	57	57	61	58	62	61	3.8	1.8	1.51	1.33	1.48	1.54	1.36	1.5	0.11
	TAM+DOC	24 h	73	75	70	71	73	68	72	2	2	1.86	1.57	2	1.76	1.45	1.74	0.18
		48 h	48	74	63	59	66	70	63	6.6	1.19	1.8	1.4	1.41	1.61	1.42	1.47	0.16
		96 h	80	69	71	81	49	59	68	9.4	2	1.76	1.7	2	1.52	1.33	1.71	0.2
	TAM+DOX	24 h	75	63	56	58	62	58	62	4.6	1.81	1.53	1.34	1.5	1.43	1.22	1.47	0.14
		48 h	59	56	60	68	74	63	63	5.1	1.68	1.47	1.47	1.65	1.71	1.34	1.55	0.13
		96 h	76	44	57	64	41	57	57	9.3	1.7	1.36	1.31	1.61	1.19	1.33	1.41	0.16
ZR751 ER+/PR+/HER2−	CTRL		50	45	56	46	58	47	50	4.4	1.19	1.24	1.13	1.14	1.32	1.16	1.2	0.06
	TAM	24 h	62	46	45	61	58	43	53	7.8	1.4	1.15	1.07	1.43	1.47	1.45	1.33	0.15
		48 h	47	49	49	59	64	53	54	5.3	1.34	1.16	1.19	1.51	1.41	1.22	1.3	0.12
		96 h	58	69	45	61	63	55	59	5.8	1.41	1.61	1.19	1.52	1.5	1.25	1.41	0.13
	DOC	24 h	69	72	63	62	64	69	67	3.5	1.84	1.75	1.35	1.41	1.7	1.6	1.61	0.16
		48 h	70	52	66	61	62	53	61	5.4	1.63	1.32	1.54	1.55	1.48	1.3	1.47	0.11
		96 h	68	66	60	63	61	56	62	3.3	1.61	1.63	1.03	1.55	1.62	1.46	1.48	0.16
	DOX	24 h	64	49	48	45	48	57	52	5.7	1.42	1.36	1.9	1.17	1.25	1.29	1.4	0.17
		48 h	59	44	66	67	61	50	58	7.2	1.45	1.15	1.39	1.63	1.47	1.22	1.39	0.13
		96 h	68	48	72	55	50	65	60	8.6	1.85	1.23	1.72	1.55	1.13	1.36	1.47	0.23

Table S2 Continued

Cell line	Treatments	Time	CIN						Mean CIN	SD	SDI						Mean SDI	SD
			CEP2	CEP 3	CEP8	CEP 11	CEP 15	CEP17			CEP2	CEP 3	CEP8	CEP 11	CEP 15	CEP17		
	TAM+DOC	24 h	68	55	59	47	62	59	58	4.8	1.6	1.42	1.3	1.33	1.3	1.3	1.38	0.09
		48 h	78	71	65	50	65	73	67	7	2.02	2.03	1.58	1.4	1.83	1.81	1.78	0.19
		96 h	69	63	73	62	64	69	67	3.6	1.65	1.76	1.55	1.41	1.62	1.59	1.6	0.08
	TAM+DOX	24 h	69	76	71	68	72	62	70	3.3	1.76	1.85	1.67	1.7	1.8	1.46	1.7	0.1
		48 h	74	60	67	57	65	60	64	4.8	1.88	1.33	1.67	1.51	1.56	1.36	1.55	0.15
		96 h	74	63	62	62	67	61	65	3.7	2	1.66	1.27	1.55	1.65	1.31	1.57	0.2
MDA-MB468 ER−/PR−/HER2−	CTRL		75	48	58	30	33	54	50	12.6	1.91	1.2	1.22	0.82	0.9	1.05	1.18	0.26
	TAM	24 h	79	78	65	32	73	63	65	11.6	2.03	2	1.54	1.04	1.75	1.32	1.61	0.31
		48 h	75	68	55	45	61	61	61	7.2	2	1.56	1.17	1.27	1.5	1.41	1.47	0.2
		96 h	71	57	50	31	53	61	54	9.1	1.65	1.37	1.08	0.96	1.22	1.33	1.27	0.18
	DOC	24 h	69	79	66	54	49	64	64	8	1.73	2.06	1.48	1.35	1.32	1.31	1.54	0.24
		48 h	68	67	57	44	45	56	56	7.8	2	1.54	1.31	1.28	1.18	1.35	1.43	0.22
		96 h	81	69	67	65	49	70	67	6.5	2.01	1.6	1.37	1.6	1.27	1.53	1.56	0.17
	DOX	24 h	74	56	63	31	44	45	52	12.1	1.76	1.18	1.31	1.08	1.05	1.08	1.24	0.19
		48 h	62	60	64	39	40	53	53	9	1.63	1.18	1.37	1.16	0.9	1.19	1.24	0.17
		96 h	74	46	68	58	40	60	58	9.7	1.76	1.18	1.46	1.56	1.03	1.16	1.36	0.24
	TAM+DOC	24 h	62	55	61	42	47	54	54	6	1.54	1.18	1.38	1.18	1.11	1.26	1.28	0.12
		48 h	80	62	65	34	32	63	56	15.3	2.09	1.38	1.37	0.97	0.91	1.28	1.18	0.19
		96 h	77	79	57	34	69	68	64	12.3	2	2	1.38	0.99	1.62	1.44	1.55	0.3
	TAM+DOX	24 h	80	64	62	45	30	61	57	13	2	1.28	1.33	1.46	1	1.28	1.4	0.23
		48 h	70	51	57	36	27	52	48	11.5	1.83	1.06	1.29	1.16	0.8	1.14	1.21	0.23
		96 h	78	24	58	30	24	56	45	19	1.87	1.14	1.28	0.93	0.74	1.3	1.21	0.27
BT474 ER+/PR+/HER2+	CTRL		62	69	42	66	60	65	61	7.5	1.33	1.6	0.95	1.46	1.45	1.57	1.4	0.17
	TAM	24 h	67	63	69	54	60	49	61	6	1.6	1.48	1.55	1.35	1.32	1.05	1.4	0.15
		48 h	68	74	71	57	59	73	67	7.04	1.63	1.65	1.58	1.41	1.51	1.63	1.56	0.07
		96 h	63	72	69	57	75	71	68	4.7	1.5	1.64	1.37	1.35	1.81	1.6	1.55	0.14

Table S2 Continued

Cell line	Treatments	Time	CIN						Mean CIN	SD	SDI						Mean SDI	SD
			CEP2	CEP 3	CEP8	CEP 11	CEP 15	CEP17			CEP2	CEP 3	CEP8	CEP 11	CEP 15	CEP17		
	DOC	24 h	75	70	69	59	65	73	68	4.3	1.9	1.48	1.55	1.44	1.31	1.79	1.57	0.18
		48 h	73	80	72	64	74	72	72	3.1	1.92	2.23	1.27	1.5	1.87	1.74	1.75	0.25
		96 h	81	70	70	63	73	69	72	4	2.28	1.79	1.79	1.67	1.81	1.74	1.85	0.14
	DOX	24 h	81	73	76	65	71	58	71	6.1	2.34	1.86	1.82	1.66	1.81	1.37	1.81	0.2
		48 h	74	71	69	46	67	67	66	6.5	1.9	1.69	1.61	1.2	1.67	1.46	1.6	0.17
		96 h	78	68	66	63	61	68	67	4	2.08	1.67	1.51	1.58	1.6	1.52	1.66	0.14
	HT	24 h	71	55	67	48	65	61	61	6.5	1.78	1.31	1.51	1.28	1.51	1.32	1.45	0.15
		48 h	73	70	61	66	76	57	67	5.8	1.85	1.79	1.28	1.5	2.01	1.39	1.64	0.25
		96 h	68	70	63	58	69	67	66	3.5	1.74	1.69	1.53	1.37	1.64	1.42	1.57	0.13
	TAM+DOC	24 h	73	71	69	63	71	70	70	2.3	1.74	1.63	1.48	1.39	1.82	1.64	1.62	0.12
		48 h	72	66	71	46	68	61	64	7	1.79	1.68	1.57	1.2	1.63	1.5	1.56	0.14
		96 h	73	70	53	49	68	71	64	8.6	1.84	1.71	1.33	1.35	1.69	1.46	1.56	0.18
	TAM+DOX	24 h	80	76	69	53	81	73	72	7.3	2.11	1.95	1.51	1.51	1.99	1.65	1.79	0.23
		48 h	73	71	73	56	75	68	69	4.8	1.79	1.78	1.68	1.48	1.69	1.4	1.64	0.13
		96 h	69	78	70	53	83	72	71	6.8	1.83	1.9	1.53	1.34	2.1	1.5	1.7	0.24
	TAM+HT	24 h	78	78	63	76	64	64	71	6.8	1.87	1.99	1.55	1.88	1.84	1.46	1.77	0.17
		48 h	74	74	60	62	74	67	69	5.5	1.77	1.81	1.48	1.49	1.78	1.44	1.63	0.16
		96 h	71	76	69	70	79	64	72	4	1.78	1.86	1.48	1.62	1.99	1.47	1.7	0.18
	HT+DOC	24 h	71	73	50	61	78	59	65	8.6	1.79	1.66	1.2	1.19	1.85	1.54	1.54	0.23
		48 h	76	68	65	59	64	54	64	5.3	1.81	1.56	1.28	1.52	1.5	1.37	1.51	0.12
		96 h	79	74	59	52	71	57	65	9.3	1.99	1.85	1.29	1.32	1.82	1.44	1.62	0.27
	HT+DOX	24 h	73	76	65	69	76	64	71	4.5	1.74	1.9	1.34	1.6	2	1.48	1.68	0.2
		48 h	79	78	63	65	76	64	71	6.8	2.2	1.9	1.49	1.47	1.92	1.36	1.72	0.28
		96 h	75	75	66	74	78	65	72	4.4	2	2.03	1.36	1.84	2.05	1.48	1.79	0.25
KPL4 ER−/PR−/HER2+	CTRL		48	57	43	53	57	45	51	5.1	1.18	1.37	1.27	1.3	1.5	1.24	1.31	0.08
	TAM	24 h	76	71	64	68	66	63	68	3.6	1.93	1.77	1.51	1.62	1.71	1.52	1.68	0.13

Table S2 Continued

Cell line	Treatments	Time	CIN						Mean CIN	SD	SDI						Mean SDI	SD
			CEP2	CEP 3	CEP8	CEP 11	CEP 15	CEP17			CEP2	CEP 3	CEP8	CEP 11	CEP 15	CEP17		
		48 h	79	54	76	64	50	66	65	8.8	1.94	1.09	1.79	1.55	1.24	1.46	1.51	0.25
		96 h	71	52	69	70	48	62	62	8	1.75	1.14	1.7	1.67	0.95	1.38	1.43	0.28
	DOC	24 h	75	68	79	63	69	74	71	4.6	2.1	1.9	2	1.77	1.81	1.9	1.91	0.09
		48 h	74	64	71	73	66	66	69	3.5	2.02	1.82	1.77	2	2	1.76	1.86	0.11
		96 h	74	79	65	71	72	70	72	3.1	2.03	2.1	1.71	2	2.02	1.84	2	0.12
	DOX	24 h	75	68	79	63	69	74	71	4.6	1.74	1.53	1.7	1.73	1.7	1.73	1.7	0.05
		48 h	74	64	71	73	66	66	69	3.6	1.85	1.93	1.78	1.83	1.99	1.81	1.87	0.06
		96 h	74	79	65	71	72	70	72	3.1	1.94	1.75	1.73	1.84	1.96	1.91	1.86	0.08
	HT	24 h	67	70	64	68	65	64	66	2	1.61	1.75	1.74	1.66	1.76	1.6	1.69	0.06
		48 h	66	74	66	69	62	70	68	3.1	1.63	2.09	1.63	1.72	1.89	1.75	1.79	0.14
		96 h	67	67	66	66	67	68	67	0.5	1.81	1.66	1.6	1.65	1.78	1.73	1.7	0.07
	TAM+DOC	24 h	83	73	67	84	67	68	74	6.5	2.41	1.92	1.86	2.35	1.77	1.81	2.02	0.24
		48 h	73	86	64	79	86	84	79	6.7	1.88	2.58	1.67	1.95	2.5	2.35	2.16	32
		96 h	72	66	72	77	64	63	69	4.6	1.93	1.86	1.86	2.24	1.92	1.84	1.94	0.1
	TAM+DOX	24 h	74	76	64	72	75	75	73	3.1	1.91	2.1	1.94	1.96	2	1.87	1.96	0.06
		48 h	39	85	66	63	83	75	69	12.5	1.07	2.53	1.44	1.52	2.46	1.87	1.81	0.47
		96 h	74	83	65	71	80	79	75	5.3	2.03	2.43	1.71	1.89	2.33	1.97	2.06	0.21
	TAM+HT	24 h	76	70	60	62	75	73	69	5.5	1.9	1.95	1.62	1.6	2.03	1.82	1.82	0.14
		48 h	64	89	64	70	83	84	76	9.6	1.63	2.8	1.86	2	2.62	2.6	2.25	0.42
		96 h	75	86	73	69	73	73	75	3.7	1.83	2.5	1.66	1.78	2	2	1.96	0.21
	HT+DOC	24 h	71	73	66	69	74	74	71	2.5	2.01	2.18	1.83	2.05	2.23	2.21	2.09	0.12
		48 h	76	72	72	73	63	63	69	4.5	2.16	2.08	1.82	2	2.01	1.87	2	0.1
		96 h	70	79	71	62	75	68	71	4.1	1.73	2.32	1.65	1.76	2.33	1.61	2	0.28
	HT+DOX	24 h	61	70	59	61	67	67	64	3.8	1.82	2	1.6	1.65	1.83	1.87	1.8	0.11
		48 h	67	71	58	65	72	72	68	4.1	1.74	1.77	1.62	1.43	2.14	1.73	1.74	0.15
		96 h	65	74	61	63	77	65	68	5.3	1.5	2	1.42	1.49	2.1	1.91	1.74	0.27

CIN, chromosomal instability; CTRL, control; DOC, docetaxel; DOX, doxorubicin; HT, herceptin; SD, standard deviation; SDI, Shannon diversity index; TAM, tamoxifen.
